# Subgenomic flavivirus RNA (sfRNA) associated with Asian lineage Zika virus identified in three species of Ugandan bats (family Pteropodidae)

**DOI:** 10.1038/s41598-021-87816-5

**Published:** 2021-04-16

**Authors:** Anna C. Fagre, Juliette Lewis, Megan R. Miller, Eric C. Mossel, Julius J. Lutwama, Luke Nyakarahuka, Teddy Nakayiki, Robert Kityo, Betty Nalikka, Jonathan S. Towner, Brian R. Amman, Tara K. Sealy, Brian Foy, Tony Schountz, John Anderson, Rebekah C. Kading

**Affiliations:** 1 Colorado State University, Fort Collins, CO USA; 2 Centers for Disease Control and Prevention, Fort Collins, CO USA; 3 Uganda Virus Research Institute, Entebbe, Uganda; 4 Makerere University, Kampala, Uganda; 5 Centers for Disease Control and Prevention, Atlanta, GA USA

**Keywords:** Viral epidemiology, Viral reservoirs, Viral transmission, Ecological epidemiology, Molecular ecology

## Abstract

Serological cross-reactivity among flaviviruses makes determining the prior arbovirus exposure of animals challenging in areas where multiple flavivirus strains are circulating. We hypothesized that prior infection with ZIKV could be confirmed through the presence of subgenomic flavivirus RNA (sfRNA) of the 3′ untranslated region (UTR), which persists in tissues due to XRN-1 stalling during RNA decay. We amplified ZIKV sfRNA but not NS5 from three experimentally-infected Jamaican fruit bats, supporting the hypothesis of sfRNA tissue persistence. Applying this approach to 198 field samples from Uganda, we confirmed presence of ZIKV sfRNA, but not NS5, in four bats representing three species: *Eidolon helvum* (n = 2), *Epomophorus labiatus* (n = 1), and *Rousettus aegyptiacus* (n = 1). Amplified sequence was most closely related to Asian lineage ZIKV. Our results support the use of sfRNA as a means of identifying previous flavivirus infection and describe the first detection of ZIKV RNA in East African bats.

## Introduction

Zika virus (ZIKV), a primarily sylvatic virus known to circulate between mosquitoes and non-human primates^1,2^, has emerged in the last decade as a pathogen of global health importance. ZIKV is a positive-sense RNA virus of the viral family *Flaviviridae*, and is transmitted to vertebrates by mosquitoes in the genus *Aedes*^1^, though it can also be transmitted between vertebrates sexually and vertically^3,4^. As efforts have been made to better characterize ZIKV pathogenesis for preventive and therapeutic purposes, knowledge gaps remain surrounding the epidemiology and ecology of the virus^5,6^. While non-human primates and mosquitoes are already implicated in the transmission cycle of ZIKV, the role of other vertebrates in sylvatic transmission, particularly in areas of introduction where non-human primates are absent, must be considered. Bats (order *Chiroptera*) have been increasingly implicated as reservoirs for many medically important groups of viruses including lyssaviruses, henipaviruses, filoviruses and coronaviruses^7–13^. However, field studies investigating their role as reservoirs of arboviruses including ZIKV are lacking^14^. A better understanding of the sylvatic ecology of ZIKV could provide insights into viral evolution and host adaptation, in addition to informing risk analysis and strategies to prevent viral transmission.

The MR766 strain of ZIKV was originally isolated from a sentinel rhesus macaque (*Macaca mulatta*) in the canopy of Zika forest in 1947^2^, where bats also reside. While the importance of canopy-dwelling mosquitoes in Zika forest (Uganda) was initially recognized in the context of yellow fever virus (YFV) transmission, Haddow et al. (1964) later reported numerous isolations of ZIKV from the arboreal sylvan mosquito, *Aedes (Stegomyia) africanus*^1^. We hypothesized that if sylvatic ZIKV is being transmitted by arboreal mosquito vectors in the forest canopy among non-human primates, then bats congregating at this forest stratification to roost and/or feed on fruits would also be exposed to feeding mosquitoes. Other studies have demonstrated cohabitation of bats and mosquitoes in caves, and mosquito bloodmeals taken from frugivorous bats have been confirmed in Uganda^15^.

Early studies examined the potential for ZIKV MR766 to infect experimentally-inoculated bats (African straw-colored fruit bat (*Eidolon helvum*)*,* Angolan rousette bat (*Lissonycteris angolensis*)*,* and Egyptian rousette bat (*Rousettus aegyptiacus*)) and assessed seroprevalence in free-ranging bats (Little free-tailed bat (*Chaerephon pumila*), African straw-colored fruit bat, Angolan free-tailed bat (*Mops condylurus*), and Egyptian rousette bat from Uganda^16,17^. A more recent field study indicated the widespread presence of flavivirus antibodies in both frugivorous and insectivorous Ugandan bats^18^. Experimental infection of Jamaican fruit bats (*Artibeus jamaicensis*) with the strain causing the 2015–2016 epidemic (ZIKV PRVABC59) resulted in seroconversion and the detection of viral nucleic acid in multiple tissues^19^, suggesting the potential for New World fruit bats to contribute to the sylvatic transmission of ZIKV PRVABC59 in the Americas. Additional studies describing flaviviruses in bats have been reviewed in detail elsewhere, and suggest their potential to contribute to sylvatic flavivirus transmission owing to overlapping habitat with mosquitoes as well as bloodmeal data from mosquitoes known to vector ZIKV^5^.

Kading et al. (2018) demonstrated the presence of non-specific flavivirus antibodies in both frugivorous and insectivorous Ugandan bats, yet no nucleic acid was detected in the spleens of seropositive bats when tested with pan-flavivirus primers targeting NS5^18,20^. It is likely that the bats had been infected in the past, and viral nucleic acid was no longer present due to nucleic acid degradation by host cell enzymes. mRNA degradation in cells is a well-characterized phenomenon that regulates steady-state RNA levels^21^. RNA viruses have evolved mechanisms to combat degradation by these enzymes and protect viral nucleic acid for replication and other purpose^22^. Among these mechanisms, subgenomic flaviviral RNA (sfRNA) in the 3′ untranslated region (UTR) is known to persist at higher levels in host tissue than genomic RNA, due to its ability to stall exoribonuclease-1 (XRN-1) on the complex hairpin structures characteristic of viral 3′ sequences. This stalling results in incomplete degradation of viral transcripts and subsequent accumulation of these short, subgenomic sequences (sfRNA) in cells and tissue^23–25^. For this reason, ZIKV sfRNA was selected as a detection target to confirm the past exposure of wild-caught bats to ZIKV, operating under the hypothesis that while the majority of viral RNA will have been degraded, these residual fragments of RNA would provide a longer window of opportunity to detect past viral infection.

This study provides the first published report of ZIKV RNA in free-ranging bats, representing a strain that most closely aligns with strains in the Asian lineage. It also describes the application of sfRNA as a target for detection of residual viral RNA in free-ranging wildlife.

## Methods

### Preparation of positive controls for molecular testing

ZIKV strains MR766, PRVABC59, and DakAR41525 were separately propagated on Vero cells (ATCC CCL-81). Cell supernatant was harvested 72 hpi, and RNA extraction was performed using Trizol. Due to undetectable RNA concentration, the maximum input volume of 11 µL was used for cDNA generation using the SuperScript IV First-Strand Synthesis System with random hexamers (Thermo Fisher Scientific, Waltham, MA, United States). A ten-fold dilution series of RNA was generated for each strain to validate detection of phylogenetically divergent strains of ZIKV using our primer set. For all molecular assays, 3 µL of 10^−3^ of MR766 was used experimentally as the positive control. Propagation of ZIKV was conducted under CSU biosafety protocol 17-059B.

### Infection protocol, RNA Extraction, and cDNA synthesis for A129 mice and Jamaican fruit bats

All animal studies were carried out in accordance with ARRIVE guidelines and all procedures approved by and carried out under the Colorado State University Institutional Animal Care and Use Committee (protocol 15-6677AA). Three sub-adult male A129 mice and three female Jamaican fruit bats (*Artibeus jamaicensis)* were obtained from their respective breeding colonies at Colorado State University. Mice were subcutaneously inoculated with 1 × 10^3^ PFU supernatant from PRVABC59-infected Vero cells, and bats were subcutaneously inoculated with 7.5 × 10^5^ PFU supernatant from Vero cells infected with one of three strains (either PRVABC59, MR766, or DakAR41525; one strain per individual). Mice were euthanized at 7 days post-infection (dpi). The bat infected with ZIKV strain MR766 was euthanized at 28 dpi, while the two bats infected with strains PRVABC59 and DakAR41525 were euthanized at 45 dpi to provide a broader of time window in which to characterize sfRNA persistence. Organs and blood were harvested and placed into DMEM supplemented with 1% penicillin/streptomycin (Thermo Fisher Scientific, Waltham, MA, United States) and 10% FBS (Atlas Biologicals, Fort Collins, CO, United States) and stored at − 80 °C until RNA extraction using the Mag-Bind Viral DNA/RNA 96 kit (Omega Bio-Tek Inc., Norcross, GA, United States) on the KingFisher Flex Magnetic Particle Processor (Thermo Fisher Scientific, Waltham, MA, United States). RNA was eluted in 30 µL nuclease-free water.

### Droplet digital PCR (ddPCR) to detect ZIKV sfRNA

To detect ZIKV sfRNA, primers were designed to target the 3′ UTR of multiple strains of ZIKV according to recommended ddPCR primer design guidelines, resulting in an amplicon 123 bp in length (F: TTCCCCACCCTTYAATCTGG and R: TGGTCTTTCCCAGCGTCAAT). Each reaction consisted of 50 ng cDNA, 125 nM foward primer, 125 nM reverse primer, and 10 µL QX200 ddPCR EvaGreen Supermix (Bio-Rad Laboratories, Hercules, CA, United States). Following reaction preparation, 20 µL of reaction and 60 µL of QX200 Droplet Generation Oil for EvaGreen (Bio-Rad Laboratories, Hercules, CA, United States) were loaded into a DG8 Cartridge for droplet generation in the QX200 Droplet Generator (Bio-Rad Laboratories, Hercules, CA, United States). Following droplet generation, plates were sealed in the PX1 PCR Plate Sealer (Bio-Rad Laboratories, Hercules, CA, United States). PCR was performed on a T100 Thermal Cycler (Bio-Rad Laboratories, Hercules, CA, United States), using the following cycling parameters: 95 °C for 5 min, 40 cycles of 95 °C for 30 s followed by 57.5 °C for 1 min, 4 °C for 5 min, 90 °C for 5 min, and held at 4 °C until reading the plate. Plates were read on the QX200 Droplet Reader (Bio-Rad Laboratories, Hercules, CA, United States). Analysis was performed by two individuals using QuantaSoft Software (Bio-Rad Laboratories, Hercules, CA, United States) to determine results.

Gradient PCR was performed to identify the optimal annealing temperature, resulting in selection of 57.5 °C (Fig. S1). At this annealing temperature, the ddPCR reaction using the 3′ UTR primers successfully amplified ZIKV strains MR766, DakAR41525, and PRVABC59 (Fig. S2). As an additional and more biologically relevant sample type, 50 ng cDNA from the organs of A129 mice experimentally infected with ZIKV PRVABC59 were tested using this same assay; successful ZIKV sfRNA amplification was obtained from mouse kidney and spleen (Fig. S2). Blood and tissue samples from the three female Jamaican fruit bats were tested in duplicate on the QX200 Droplet Digital (ddPCR) System (Bio-Rad Laboratories, Hercules, CA, United States) using the ZIKV sfRNA primers as described above.

### Testing of archived samples from free-ranging Ugandan bats

This study utilized archived tissue samples from bats previously captured in Uganda from 2009 to 2013^18,26^ (Table 1). Bats were captured using harp traps or mist nets, identified using a field guide specific to East African bats, and placed in holding bags prior to anesthesia via halothane and euthanasia by cervical dislocation^27^. This study used historic archived samples from a previous study, in which all bat captures and sampling were conducted under the approval of CDC IACUC protocols 1731AMMULX and 010-015 and carried out according to ARRIVE guidelines. RNA was extracted from frozen tissue homogenates (spleen, and in some cases both spleen and liver separately) using the MagMax 96 total RNA isolation kit (Applied Biosystems, Foster City, CA, United States), and cDNA generation was performed as above. To confirm RNA integrity via amplification of a housekeeping gene, we used previously published primers demonstrated to amplify GAPDH from two Old World bat species (black flying fox and Egyptian rousette bat) and one New World bat species (common vampire bat) (F: GTCGCCATCAATGACCCCTTC and R: TTCAAGTGAGCCCCAGCC)^31^. For samples with undetectable RNA concentration on the Qubit RNA HS assay, 6 µL cDNA was used as input. ddPCR was performed as above, except that an annealing temperature of 60˚C was used. Plates were read as above, and only samples deemed ‘suspect’ or ‘positive’ for GAPDH amplification were subjected to ddPCR testing with ZIKV sfRNA (3′ UTR). For these samples, the same volume of input cDNA was used to test for the presence of ZIKV sfRNA in duplicate; results were analyzed by two individuals.

**Table 1:** All bat species and trap sites collected from 2009 to 2013^18,26^.

Location	Latitude	Longitude	BAT SPECIES								
			ROAE	CHPU	EIHE	EPLA	HIRU	LIAN	MOCO	SCHI	TOTAL
Python cave	− 0.26667	30.05000	71	–	–	–	–	–	–	–	71
Kasokero cave	− 0.34214	31.96627	55	–	–	–	3	–	–	–	58
Tutum cave	1.28333	34.46667	45	–	–	–	–	–	–	–	45
Banga, Nakiwogo	0.08333	32.45000	–	28	–	–	–	–	26	–	54
Kawuku	0.13487	32.53392	–	63	–	51	–	–	–	–	114
Kisubi	0.11826	32.53017	–	6	–	–	–	–	–	–	6
Namasuba	0.29778	32.81861	–	17	–	–	–	–	–	–	17
Zika forest	0.11667	32.53333	–	2	–	–	–	–	1	1	4
Bugonga	0.05000	32.46667	–	–	7 (5)	–	–	–	–	–	7
Jinja	0.41667	33.20000	-	-	8	–	-	–	–	–	8
Buwaya Lugonjo	0.08333	32.43333	–	–	–	23	–	–	–	–	23
Kasange	0.15000	32.40000	–	–	–	4	–	–	–	–	4
Kikaaya	0.37017	32.58932	–	–	–	16 (1)	–	–	–	–	16
Kapkwai cave	1.33333	34.41667	–	–	–	–	3	9	–	–	12
Species sum			171	116	15 (5)	94 (1)	6	9	27	1	439 (6)

Numbers in parentheses (for samples from Bugonga and Kikaaya) indicate the number of individuals from which both spleen and liver were collected and analyzed separately (n = 6). Owing to some bats having two organs sampled, we tested a total of 445 samples from 439 bats. (Species codes as follows: ROAE = *Rousettus aegyptiacus*, CHPU = *Chaerephon pumila*, EIHE = *Eidolon helvum*, EPLA = *Epomophorus labiatus*, HIRU = *Hipposideros ruber*, LIAN = *Lissonycteris angolensis*, MOCO = *Mops condylura*, SCHI = *Scotoecus hindei*).

### Sequence confirmation

To confirm specific amplification of GAPDH sequence for each of the 8 Old World species, the same primers were used in a conventional PCR assay using GoTaq HotStart Polymerase (Promega corporation, Madison, WI, United States). Cycling parameters were as follows: 95 °C for 2 min; 35 cycles of 95 °C for 1 min, 57.5 °C for 1 min, and 72 °C for 30 s; followed by 72 °C for 5 min and samples were held at 4 °C until being analyzed for the presence of a 248-bp amplicon via gel electrophoresis. Amplicons were verified by Sanger sequencing (GENEWIZ, Inc., South Plainfield NJ, United States). Results obtained from Sanger sequencing were subjected to quality analysis prior to aligning forward and reverse reads, and the consensus read was subjected to a BLAST search.

### Confirmation of ZIKV sfRNA ddPCR results in Ugandan bat samples using conventional PCR and sequencing

Samples deemed ‘suspect’ via screening on the ddPCR system with ZIKV 3′ UTR primers were subjected to additional PCR and Sanger sequencing using the same primer set targeting the 3′ UTR of ZIKV. ZIKV strain MR766 was used as a positive control in these assays. Samples were considered ‘suspect’ if (1) the automatically-defined threshold yielded ≥ 1 positive droplet in the same 1D amplitude as the positive control cDNA (ZIKV MR766) or (2) the negative droplet populations existed in the same 1D amplitude region of positive control droplets and thus, precluded the ability to differentiate positive and negative populations. The cDNA from these samples was amplified using the GoTaq HotStart system (Promega corporation, Madison, WI, United States), with each reaction consisting of 50 ng cDNA, 25 µL GoTaq HotStart Master Mix, 400 nM forward primer, 400 nM reverse primer, and 1 M Betaine. Cycling parameters were as follows: 95 °C for 2 min; 35 cycles of 95 °C for 1 min, 57.5 °C for 1 min, and 72 °C for 30 s; followed by 72 °C for 5 min and samples were held at 4 °C until being analyzed for the presence of a 123-bp amplicon via gel electrophoresis. Positive samples were verified by Sanger sequencing (GENEWIZ, Inc., South Plainfield NJ, United States). Results obtained from Sanger sequencing were subjected to quality analysis prior to BLAST search and subsequent alignment of forward and reverse reads with the 3′ UTR of ZIKV MR766 in Geneious v11.1.5 (www.geneious.com).

### Comparison of detection sensitivity between sfRNA and NS5 in field-caught samples

The four samples from which ZIKV sfRNA was amplified were subjected to cPCR amplification with GoTaq HotStart MasterMix as described above and primers designed for this study targeting NS5 from MR766, PRVABC59, and DakAR41525 in order to compare detection sensitivity (F: TGC CGC CAC CAA GAT GAA CT, R: CAT TCT CCC TTT CCA TGG ATT GAC C). Cycling parameters were as follows: 95 °C for 2 min; 35 cycles of 95 °C for 1 min, 57.5 °C for 1 min, and 72 °C for 30 s; followed by 72 °C for 5 min and samples were held at 4 °C. cDNA from ZIKV MR766 was used as a positive control. Results were sent for Sanger sequencing if a band was present. All methods in this study were carried out in accordance with relevant guidelines and regulations.

## Results

### Confirmatory PCR and Sanger sequencing of samples from bats experimentally infected with ZIKV

Experimentally challenged bats were screened for ZIKV sfRNA using the droplet digital PCR (ddPCR) platform, and then confirmed with conventional PCR (cPCR). Successful amplification and Sanger sequencing of ZIKV 3′UTR sfRNA, but not NS5, from multiple organs (brain, heart, lung, spleen, kidney, bladder, and uterus) and blood from Jamaican fruit bats subcutaneously inoculated with ZIKV (Table 2) further supports our hypothesis that sfRNA is a more sensitive detection target than NS5 due to XRN1 stalling.

**Table 2:** Jamaican fruit bats from which ZIKV sfRNA was amplified and sequenced (n = 3).

	MR766	PRVABC59	DakAR41525
	(28dpi)	(45dpi)	(45dpi)
Brain		X	X
Heart	X		X
Lung	X		X
Liver			
Spleen	X		
Kidney	X		X
Bladder	X		X
Ovary			X
Uterus	X	X	
Blood	X	X	

One animal was inoculated with each of three strains of ZIKV and euthanized at varying timepoints (e.g. female bat inoculated with MR766 euthanized at 28 dpi).

### Detection and Sanger sequencing of ZIKV RNA in field-caught bat samples

**Figure 1:**
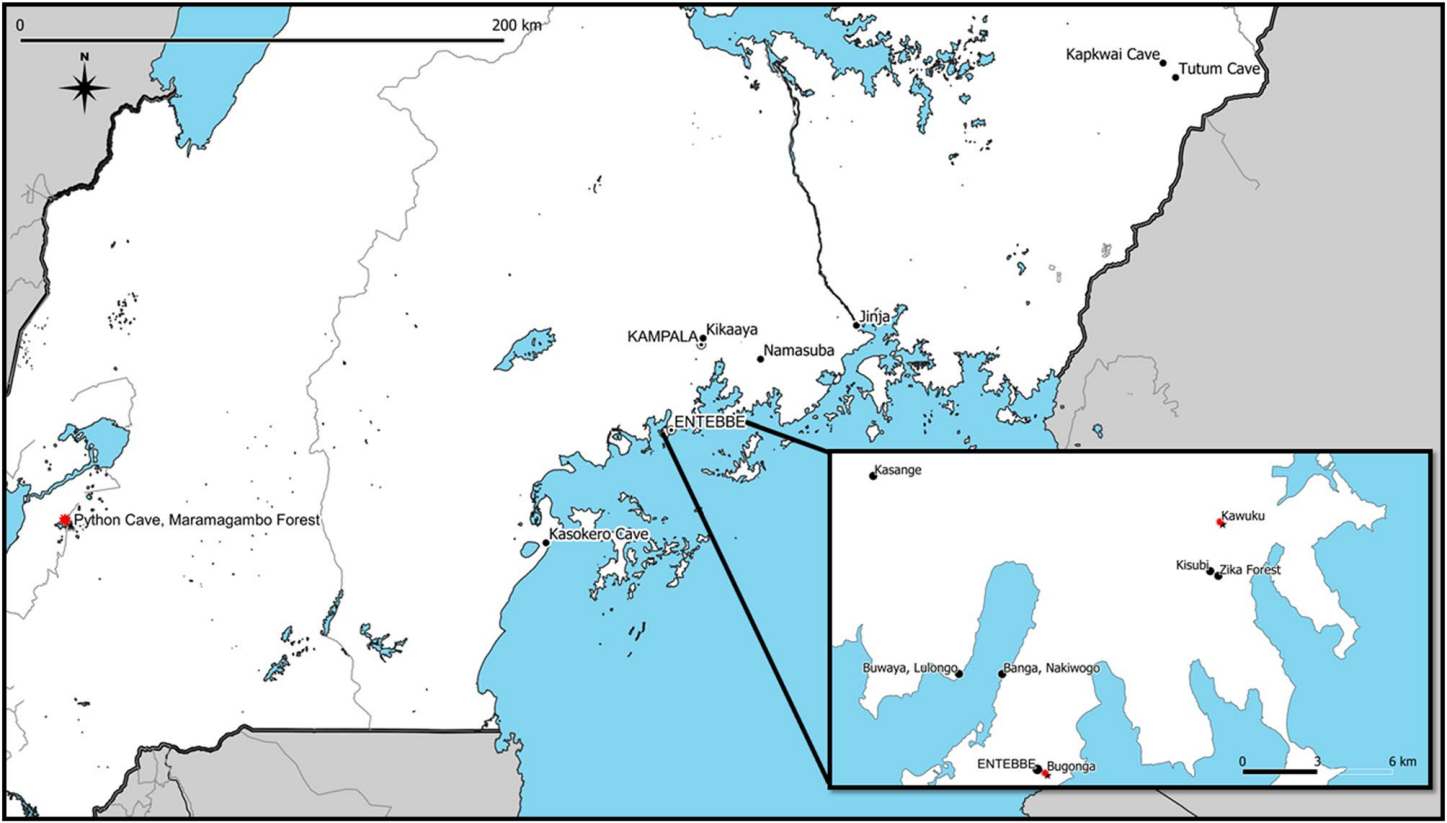
Map of Uganda, with trap sites indicated by black circles and positive sites indicated by red stars (Python Cave, Maramagambo Forest: Egyptian rousette bat, 2009 (n = 1); Kawuku: Ethiopian epauletted fruit bat, 2013 (n = 1); Bugonga: African straw-colored fruit bat, 2011 (n = 2)). Map was created using QGIS 3.4.2 (www.qgis.org).

RNA quality was confirmed by amplification of GAPDH in 198/445 archived samples, and 49/198 of these resulted in suspect results using ddPCR and were subjected to confirmatory cPCR. Of these, four of the 49 field samples from three species of bats (African straw-colored fruit bat (n = 2; Bugonga), Egyptian rousette bat (n = 1; Python cave), and Ethiopian epauletted bat (*Epomophorus labiatus*) (n = 1; Kawuku)) at three separate sites throughout Uganda were confirmed positive for ZIKV RNA by cPCR and Sanger sequencing (123-bp amplicon) (Fig. 1, S3-8). Sequences were deposited in Genbank. Sequence alignment suggest that the sfRNA detected is most closely related to Asian lineage ZIKV (Table 3).

**Table 3:** Nucleotides present at various positions in 3′ UTR indicating that sequences derived from four wild-caught bats align most closely to the Asian strain of ZIKV.

Numbered according to PRVABC59 (MH158237)	10,636	10,637	10,645	10,652	10,654	10,658	10,664
PRVABC59 (MH158237)	**T**	**C**	**G**	**A**	**A**	**G**	**A**
DakAR41525 (KU955591)	**T**	**T**	**A**	**G**	**C**	**A**	**A**
MR766 (AY632535)	**C**	**T**	**A**	**A**	**C**	**G**	**A**
*E. helvum* (bat 87, MT482106)	T	C	G	A	A	G	G
*E. helvum* (bat 89, MT482107)	T	C	G	A	A	N	A
*E. labiatus* (bat 380, MT482108)	T	C	G	A	A	G	A
*R. aegyptiacus* (bat 1354, MT482109)	T	C	G	A	A	G	A

The significance of the bold was to indicate that the nucleotides were associated with reference sequence/accessions pulled from GenBank we used for our multiple alignment.

Position are numbered based on PRVABC59 (MH158237). For each sample, bat species, identification number, and Genbank accession number are provided.

## Discussion

We confirmed the presence of 3′ UTR ZIKV RNA from free-ranging bats across Uganda. Targeting the viral 3′UTR represents a novel and sensitive approach for detection of residual flavivirus RNA in previously-infected animals. NS5 is degraded by the XRN-1 exonuclease; therefore, we hypothesized that samples positive for ZIKV 3′UTR sfRNA would be negative for NS5^26^, which was true in both experimentally-inoculated bats as well as naturally-infected bats from Uganda. The four bats from which ZIKV RNA was amplified comprised three species (African straw-colored fruit bat (n = 1), Egyptian rousette bats (n = 2), and Ethiopian epauletted fruit bat (n = 1)), all in family Pteropodidae, captured from three separate locations across Uganda, between 2009 and 2013 (Fig. 1). The viral 3′UTR sequence from all four of these bats is most closely aligned with Asian lineage ZIKV (Table 2, Fig S4). Recent reports describe detection of Asian lineage ZIKV in human cases from Cabo Verde and Angola, and genomic analysis estimates that the first introduction of Asian lineage ZIKV to Angola was between July 2015 and 2016^28^. These findings corroborate serologic evidence of flavivirus circulation within Ugandan fruit bat populations and suggest perhaps an earlier introduction of Asian lineage ZIKV to Africa than previously thought or genetic divergence of ZIKV strains prior to the eastward spread of ZIKV to Malaysia^18,29^. However, owing to limitations surrounding the length of the sfRNA amplicon obtained, further investigation and additional surveillance in wildlife and mosquito species in regions is warranted.

The natural infection of ZIKV among diverse frugivorous bat species is intriguing and suggests a more widespread exposure of wildlife to sylvatic arboviruses than currently understood. Cave-dwelling Egyptian rousette bats naturally inhabit forested areas, whereas Ethiopian epauletted fruit bats and African straw-colored flying foxes are more adapted to disturbed environments, dwelling in man-made structures and tall trees, respectively. The latter two species may visit forested habitats, raising questions regarding the exposure of each of these bats to mosquito vectors of Zika virus. Previously, mosquito blood meals matching Egyptian rousette and African straw-colored fruit bats have been confirmed from Maramagambo and Semliki forests, Uganda^15^. Serological detection of anti-ZIKV neutralizing and IgM antibodies in febrile human patients were later confirmed between 2014 and 2017, documenting active ZIKV circulation in Uganda and the first laboratory-confirmed human case in decades^30^. Investigation into the interactions between bats and arthropods transmitting viruses of medical importance in different ecological systems is warranted.

The use of MR766 strain as a positive control throughout the ddPCR and cPCR testing of samples from our experimentally-infected and field-caught bats rules out contamination as a possible explanation of these results. A natural SNP was also detected in the 3′UTR ZIKV sequence amplified from one of the two African straw-colored fruit bats (Table 2, Figure S4). Further, the amplification of sfRNA in the absence of NS5 amplification in both field-caught and experimentally infected bats illustrates the potential for this portion of the genome to act as a valuable and sensitive biosurveillance target, due to its stalling of XRN-1.

We describe the use of GAPDH as a proxy for RNA integrity, as we successfully amplified this gene from all eight of our field-caught bat species. These primers were adapted from equine GAPDH primers and have previously been demonstrated to amplify the GAPDH gene in two Old World bat species (Egyptian rousette bats and the black flying fox (*Pteropus alecto*)) and one New World bat species (the common vampire bat (*Desmodus rotundus*))^31,32^. This is a valuable tool for future biosurveillance efforts as it allows determination of viable RNA and subsequent prioritization of screening from a number of bat species across a broad taxonomic range.

This is the first published application of ddPCR to viral surveillance of wildlife samples, though other studies have used the tool for pathogen detection and for environmental monitoring^33–35^. Using the ddPCR platform, we amplified bat GAPDH and ZIKV RNA from field-caught bat samples, ZIKV NS5 and sfRNA in experimentally-infected mouse samples, and ZIKV sfRNA in the blood and organs of experimentally-infected bats from which we were unable to amplify NS5. However, variable levels of background fluorescence in all assays precluded us from using a standardized threshold or quantitative algorithms for defining the number of copies/µL, such as ‘ddPCRquant’ and ‘definetherain’^36,37^. Additional limitations of the study include the age and quality of the archived field-samples tested. The tissue samples were homogenized in cell culture media and frozen at − 80 °C for 6–10 years prior to this study. Only 44.4% (198/445) of the samples contained detectable levels of GAPDH by ddPCR, indicating that a majority of the samples had poor RNA integrity as a result of RNA degradation. Limitations in optimal amplicon length for the ddPCR platform resulted in 123-bp sequences for confirmation, precluding more extensive genomic analyses. Ultimately, while our assays were readily adaptable to a ddPCR platform, ddPCR did not present a significant advantage over traditional amplification methods, but rather the persistence of 3′UTR sfRNA provided a diagnostic advantage for detecting small amounts of residual viral RNA.

This study illustrates the use of sfRNA as a novel and highly sensitive biosurveillance target and lays the groundwork for future in vivo and field studies. Our results demonstrate the application of this molecular target for flavivirus biosurveillance in field samples from which coding RNA is no longer detectable and may be especially useful for samples with questionable or non-specific flavivirus serology results. Future studies should perform a comparative analysis of coding RNA and sfRNA levels over the course of infection to quantify the rate of degradation and determine the extent to which sfRNA persists and accumulates in tissues.

## Supplementary Information

Supplementary Information.

## Data Availability

All data generated or analysed during this study are included in this published article (and its Supplementary Information files). Sequences obtained from field-caught bats were deposited on Genbank (accessions MT482106-MT482109).
