# Supplementary material for: Subgenomic flavivirus RNA (sfRNA) associated with Asian lineage Zika virus identified in three species of Ugandan bats (family Pteropodidae)
Source: Sci Rep. 2021 Apr 16;11:8370. doi: 10.1038/s41598-021-87816-5 (PMC8052318; doi:10.1038/s41598-021-87816-5)
Supplement: Supplementary file 1 — Supplementary Information. [file 41598_2021_87816_MOESM1_ESM.pdf]

Title: Subgenomic flavivirus RNA (sfRNA) associated with Asian lineage Zika virus identified in three species of Ugandan bats (family Pteropodidae)

Authors: Anna C. Fagre*[1], Juliette Lewis[1], Megan R. Miller[1], Eric C. Mossel[2], Julius J. Lutwama[3], Luke Nyakarahuka[3], Teddie Nakayiki[3], Robert Kityo[4], Betty Nalikka[4], Jonathan S. Towner[5], Brian R. Amman[5], Tara K. Sealy[5], Brian Foy[1], Tony Schountz[1], John Anderson[1], Rebekah C. Kading*[1]

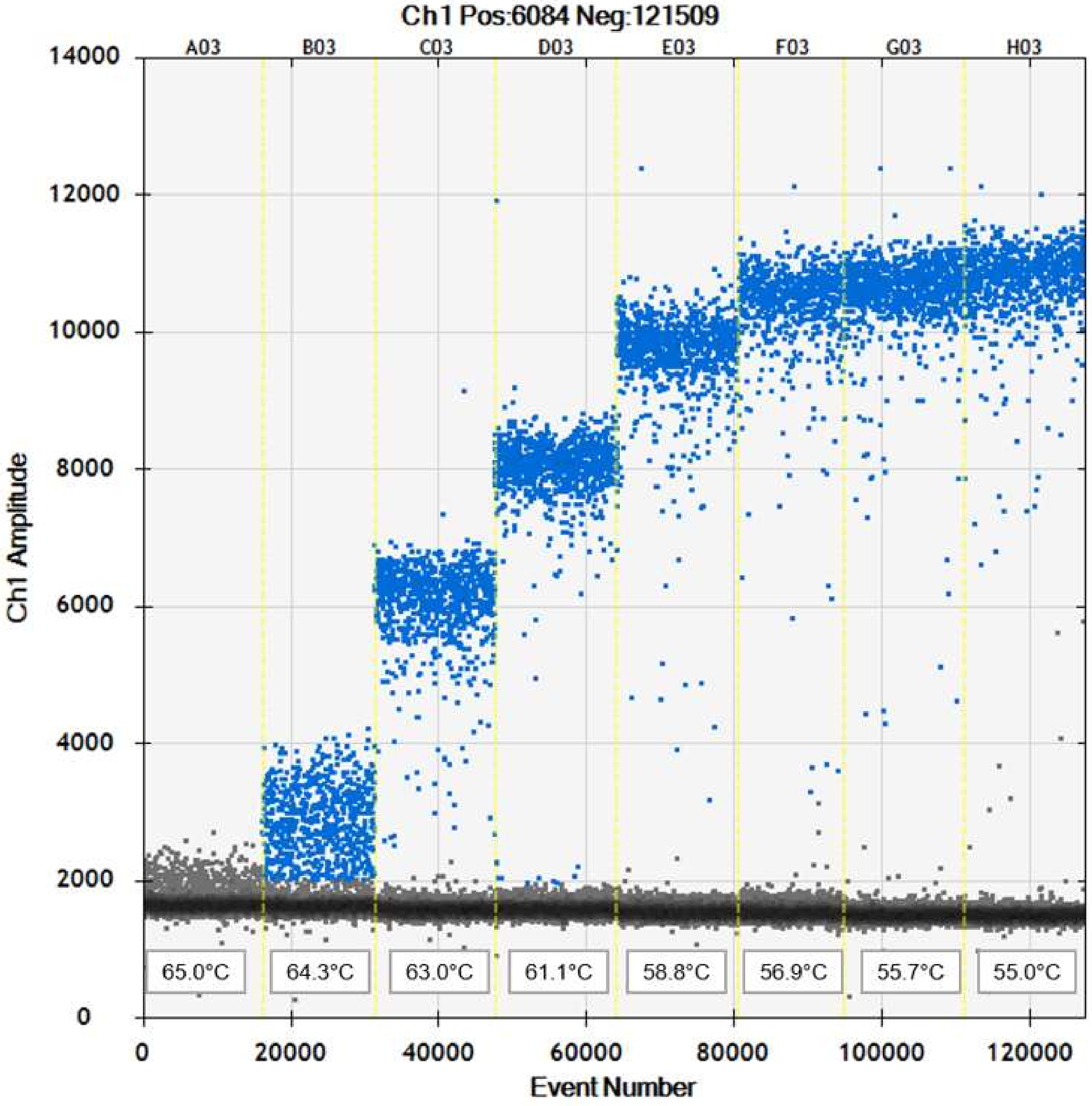

Supplementary Figure S1. A gradient PCR between 55°C-65°C with MR766 RNA was performed with best results between 56.9°C and 58.8°C. An annealing temperature of 57.5°C was selected.

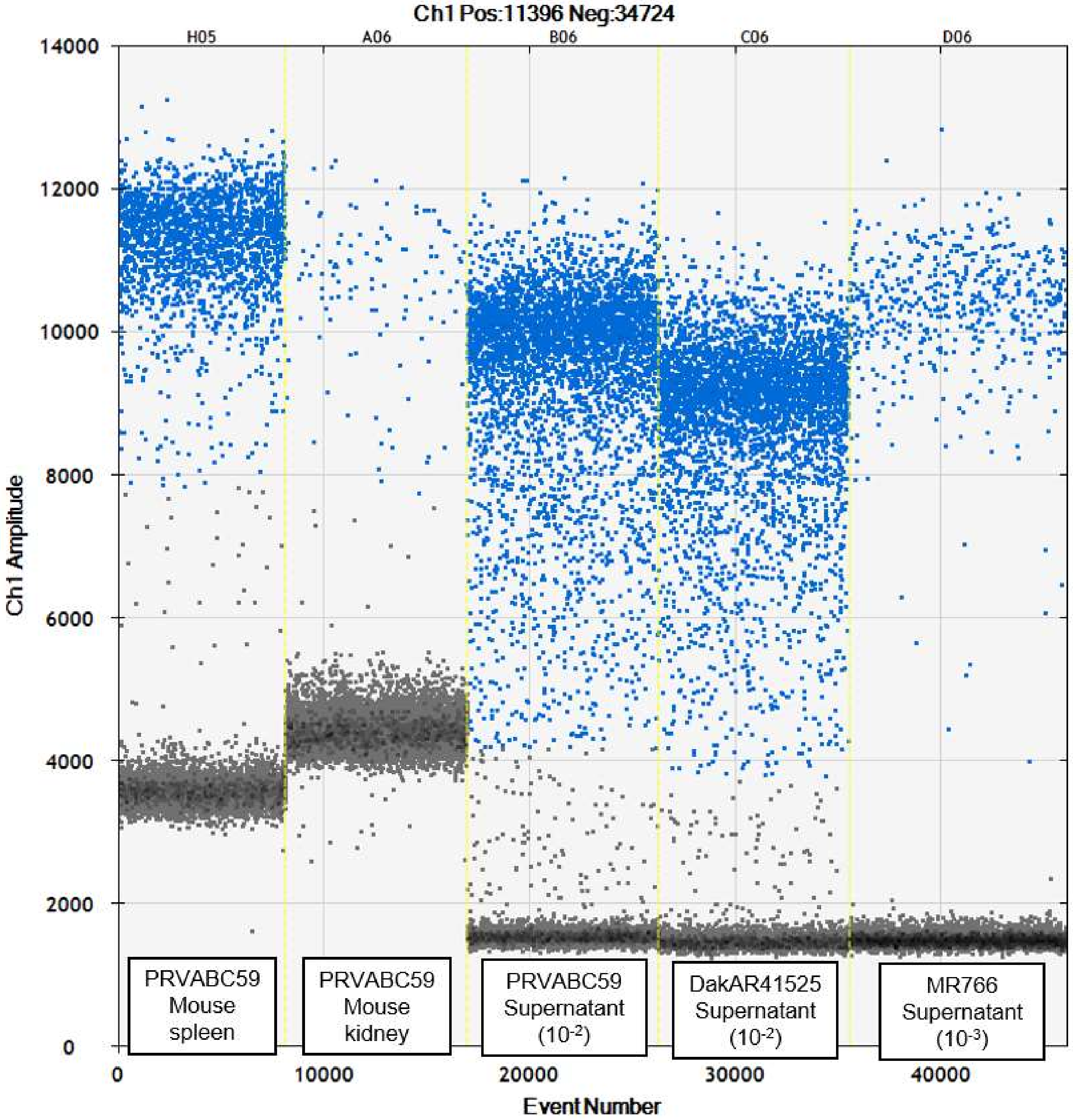

Supplementary Figure S2. Validation of the ddPCR assay to detect 1) PRVABC50 sfRNA in the organs of infected mice and 2) three genetically divergent strains of ZIKV (PRVABC59, DakAR41525, and MR766).

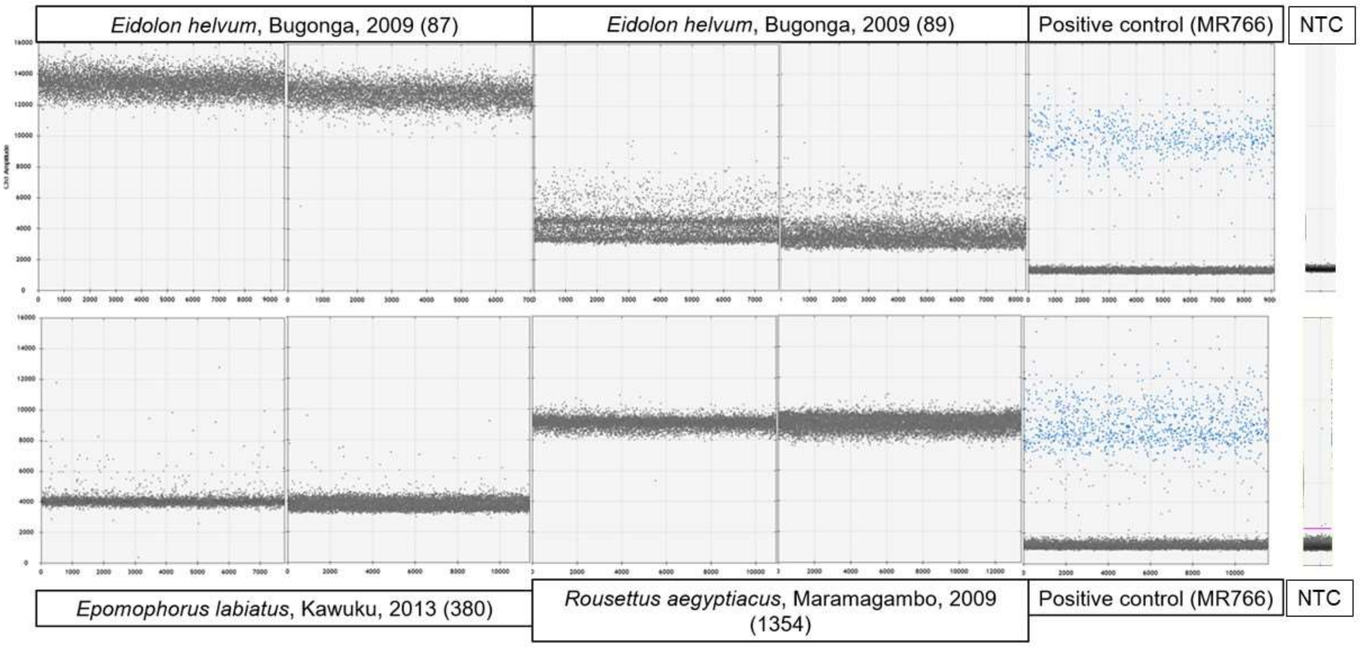

Supplementary Figure S3. ddPCR results (in duplicate) from four bats confirmed positive for ZIKV sfRNA by cPCR and Sanger sequencing with representative positive control results (RNA from MR766-infected Vero cell supernatant) and negative template control (NTC) (water).

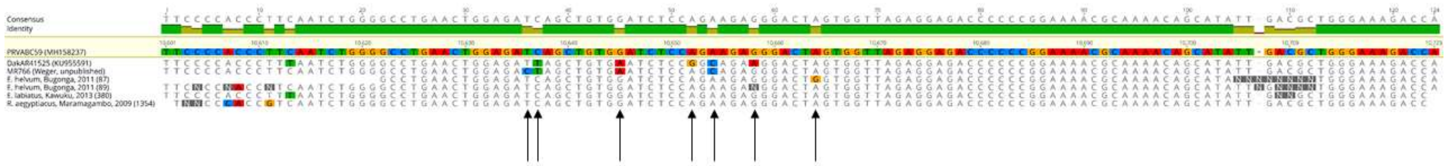

Supplementary Figure S4. Alignment of four sequences against three reference strains of ZIKV, using strain PRVABC59 as reference sequence to demonstrate SNPs (indicated by arrows). Genbank accessions for sequences used as reference strains: PRVABC59 (MH158237), DakAR 41525 (KU955591), MR766 (AY632535).

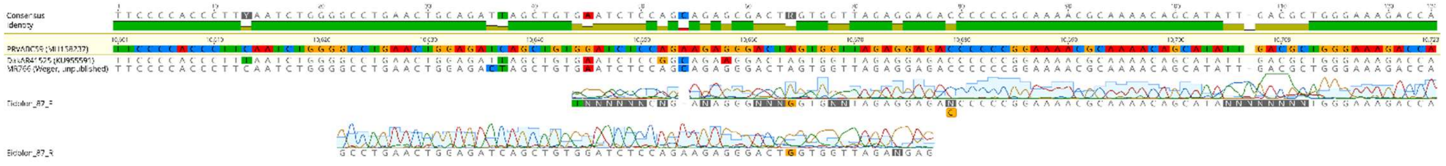

Supplementary Figure S5. See below for description.

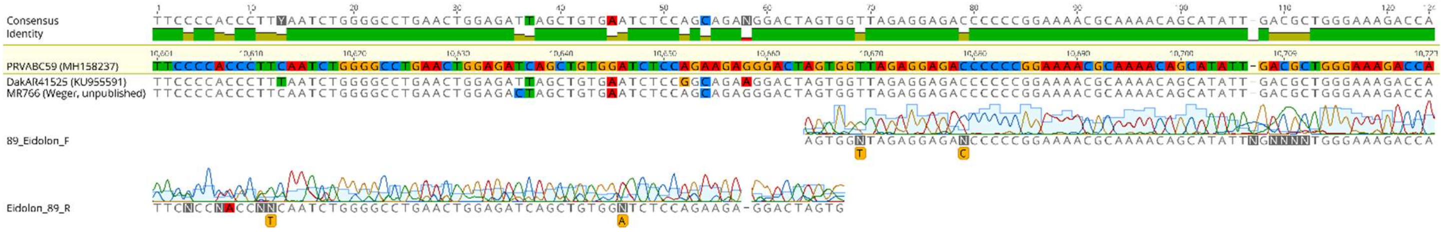

Supplementary Figure S6. See below for description.

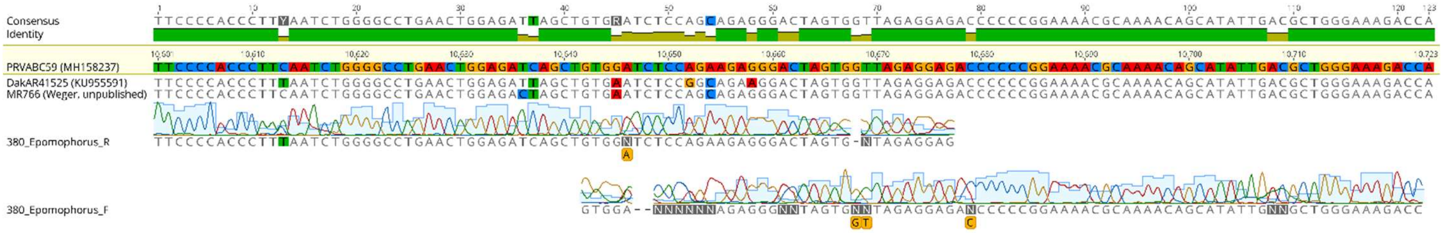

Supplementary Figure S7. See below for description.

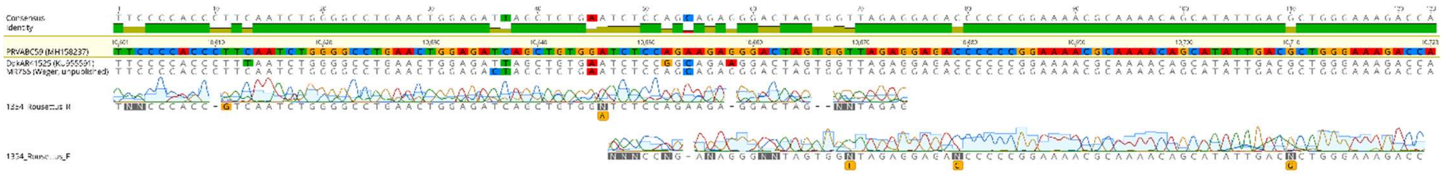

Supplementary Figure S8. See below for description.

Supplementary Figures S5-S8. Sequencing chromatograms obtained from sfRNA amplification in four bat spleens (straw-colored fruit bat (Appendix Figs. 5-6), little epauletted fruit bat (Appendix Fig 7), and Egyptian rousette bat (Appendix Fig. 8). PRVABC59 (MH158237) was used as a reference sequence. Base pair calls determined to be ambiguous upon alignment in Geneious software but resolved by authors are indicated within each chromatogram by a yellow flag, and these base pair calls are also reflected in the consensus sequences submitted to GenBank (MT482106, MT482107, MT482108, MT482109).
